# Unreliability of putative fMRI biomarkers during emotional face processing

**DOI:** 10.1016/j.neuroimage.2017.05.024

**Published:** 2017-08-01

**Authors:** C.L. Nord, A. Gray, C.J. Charpentier, O.J. Robinson, J.P. Roiser

**Affiliations:** a Institute of Cognitive Neuroscience, University College London, London, UK; b Department of Experimental Psychology, University College London, London, UK

**Keywords:** fMRI, Biomarker, Psychiatry, Emotion, Amygdala, Subgenual cingulate

## Abstract

There is considerable need to develop tailored approaches to psychiatric treatment. Numerous researchers have proposed using functional magnetic resonance imaging (fMRI) biomarkers to predict therapeutic response, in particular by measuring task-evoked subgenual anterior cingulate (sgACC) and amygdala activation in mood and anxiety disorders. Translating this to the clinic relies on the assumption that blood-oxygen-level dependent (BOLD) responses in these regions are stable within individuals. To test this assumption, we scanned a group of 29 volunteers twice (mean test-retest interval=14.3 days) and calculated the within-subject reliability of the amplitude of the amygdalae and sgACC BOLD responses to emotional faces using three paradigms: emotion identification; emotion matching; and gender classification. We also calculated the reliability of activation in a control region, the right fusiform face area (FFA). All three tasks elicited robust group activations in the amygdalae and sgACC (which changed little on average over scanning sessions), but within-subject reliability was surprisingly low, despite excellent reliability in the control right FFA region. Our findings demonstrate low statistical reliability of two important putative treatment biomarkers in mood and anxiety disorders.

## Highlights

• Reliability of neural responses to emotional faces was measured in healthy subjects.
• Robust responses were detected in the amygdalae and subgenual anterior cingulate.
• Amygdala and subgenual cingulate activation were rarely reliable over time.
• Responses in the fusiform face area were generally highly reliable.

## Introduction

Neuropsychiatric disorders are debilitating and common, and many patients fail to respond to their first treatment, with a substantial minority going on to experience chronic symptoms (Simon et al., 2006). Unlike in many branches of medicine, no useful biomarkers exist to predict treatment response in psychiatry. Instead, choosing the appropriate treatment for a specific patient typically involves a combination of clinical judgment and trial-and-error. Previous work suggests that neuroimaging may be useful in predicting treatment response to a variety of treatments for depression (Roiser et al., 2012), including cognitive behavioural therapy (Siegle et al., 2006), antidepressant medication (Brockmann et al., 2009, Keedwell et al., 2009), transcranial magnetic stimulation (Downar et al., 2013) and sleep deprivation (Ebert et al., 1991). An implicit assumption of studies using fMRI to identify treatment biomarkers is that, within an individual, the amplitude of the task-evoked blood-oxygen-level dependent (BOLD) response is stable over time (Fournier et al., 2014). That is, the signal evoked by a given task in a given region on one day should be evoked to a similar degree on another day. Such stability of measurement is essential in the interpretation of results. If a putative treatment biomarker is not reliable (at the level of the individual), it is unlikely to be useful as a predictor of clinical outcome.

Two regions in particular have emerged as strong candidates for fMRI biomarkers in depression treatment: the anterior cingulate cortex, particularly the rostral and subgenual (sgACC) portions, and the amygdala. Preliminary work in small samples has indicated that pre-treatment activation in the perigenual anterior cingulate cortex (ACC; including rostral and sgACC), particularly during emotion processing, may predict response to standard antidepressant treatment (Keedwell et al., 2009, Keedwell et al., 2010, Chen et al., 2007, Davidson et al., 2003). Pre-treatment perigenual ACC deactivation to negative stimuli has been reported to predict worse response to antidepressant treatment with two different medications, fluoxetine (Chen et al., 2007) and venlafaxine (Davidson et al., 2003). By contrast, in other studies sgACC deactivation to negative stimuli predicted *better* response to cognitive behavioural therapy (Siegle et al., 2006) and behavioural activation therapy (Dichter et al., 2010). Heightened amygdala activation during inhibitory control (Langenecker et al., 2007) and negative information processing (Siegle et al., 2006, Siegle et al., 2007) has also been reported to predict response to both antidepressant medication and cognitive behavioural therapy (CBT).

Various fMRI cognitive activation paradigms have been used to identify putative treatment biomarkers in depression, most commonly using emotional faces (Keedwell et al., 2009, Keedwell et al., 2010, Frodl et al., 2010, Fu et al., 2008a, Fu et al., 2008b). Despite robust activation when averaging across individuals, and evidence that activation can differentiate responder and non-responder groups (Keedwell et al., 2009, Keedwell et al., 2010, Langenecker et al., 2007, Fu et al., 2008b), many studies acknowledge that clinically relevant prognostic markers require high predictive accuracy at the level of the individual (Fu et al., 2013). To achieve this, the reliability of BOLD responses must be good. Several previous studies have examined the test-retest reliability of amygdala activation during emotional face processing (Johnstone et al., 2005, Sauder et al., 2013, Manuck et al., 2007, Lipp et al., 2014), reporting a range of reliabilities, ranging from near 0 (no reliability) (Plichta et al., 2012) to 0.5–0.6 (moderate–to-good reliability) (Johnstone et al., 2005, Manuck et al., 2007). However, these studies only administered a single emotion processing task, leaving open the possibility that different tasks may evoke BOLD activation with varying reliability. The reliability of sgACC activation at the subject level has never been reported to our knowledge, despite the promising preliminary results discussed above.

Therefore, we sought to measure the reliability of activation in the amygdala and sgACC across two scan days, as well as within the same session (between runs, approximately 10 min apart). Based on the higher number of reports of prediction of treatment response in depression from sgACC than amygdala activation, we hypothesised that reliability would be higher in the sgACC than in the amygdala. We employed three well-established emotional face processing tasks, which allowed us explore the relative reliability of activation they evoked. Thus, we aimed to establish whether sgACC or amygdala activation during emotional face processing could potentially serve as treatment biomarkers, as well as examining the effect of paradigm selection on inter- and intra-session reliability.

## Methods and materials

### Participants

Twenty-nine right-handed participants, 18–40 years of age (10 males) and fluent in English, were recruited through the UCL Institute of Cognitive Neuroscience subject database. Exclusion criteria included history of any neurological or mental health conditions. All participants were screened for current or past psychiatric disorders using the Mini International Neuropsychiatric Interview, version 5.0.0 (Sheehan et al., 1998). Additionally, illegal substance use was prohibited in the six weeks preceding the first MRI scan, and standard MRI safety restrictions applied. We initially recruited 35 participants, but six participants were excluded from the final analysis: four because they did not return for the second MRI scan, and two because their MRI data were lost. The final sample had a mean age of 26 (SD=6.24) and consisted of nineteen female participants. Participants were compensated £30 for both sessions. The study was approved by the UCL Departmental Research Ethics Committee (ID: fMRI/2013/005).

### Experimental paradigm

Subjects were tested on three separate days. The first day involved initial screening for psychiatric conditions and MRI contraindications and a practice session of each task, in order to decrease the novelty of the stimuli and ensure participants understood all task instructions. On the second and third testing days, which occurred 9–21 days later (mean time between scans=14.33 days (SD=2.10)), participants performed each task twice in the scanner, with the order consistent between days (including the practice day) and counterbalanced between participants. Subjects used an MRI-compatible button box to make responses during the tasks.

We employed the following three tasks: emotion identification, emotion matching, and gender classification (see Table 1). All face stimuli were sourced from the NimStim Face Stimulus Set (http://www.macbrain.org/resources.htm) (Tottenham et al., 2009).

**Table 1:** Characteristics of each task.

	Emotion matching	Emotion identification	Gender classification
Task duration *(per run)*	5:55	4:03	6:24
Task design	Blocked	Event-related	Blocked
Regressors of interest	Faces; shapes	Happy; fearful; neutral	Happy; fearful; neutral
Regressors of no interest	6 movement parameters	6 movement parameters	6 movement parameters+errors
Contrast	Faces>shapes	Faces>fixation	Faces>fixation

#### Emotion identification task (Robinson et al., 2012)

Emotional face stimuli lasting 1 s were followed by a jittered (3–5 s) centrally-presented fixation cross in a task adapted from a recent study (Robinson et al., 2012). Sixty stimuli were presented in total. There was a baseline period of fixation lasting 30 s at the beginning and end of the task. Subjects identified the emotion of the face presented as happy, fearful, or neutral, using an MRI-compatible button box with index, middle, and ring finger presses corresponding to happy, fearful, and neutral faces, respectively. Each participant was presented with a random order of male and female faces, with an equal proportion of male and female faces. We analysed the accuracy of emotion identification (data not shown) and excluded two subjects because of non-responses on more than 20 trials (indicating that they were not attending to the task); the final analysis included 27 participants.

#### Emotion matching task (Hariri et al., 2002)

Participants viewed faces displaying angry or fearful emotions, matching the emotion of a centrally-presented face to one of two alternatives presented at the bottom left and bottom right of the screen. The control condition involved matching a central shape with one of two test shapes. In both conditions, participants pressed their index finger for central stimuli that matched the bottom left stimulus, and their middle finger for central stimuli that matched the bottom right stimulus. Each participant was presented with a random order of male and female faces, with an equal proportion of male and female faces. Three blocks of shape matching and two blocks of emotion matching were performed in each run of the task, with all stimuli presented for 5 s. There were six trials in each of the five blocks, with each block lasting 30 s. We analysed the accuracy of both shape and emotion matching (data not shown). No subjects were excluded because of poor task performance (N=29).

#### Gender classification task (O’Nions et al., 2011)

Participants viewed faces displaying happy, fearful, and neutral emotions, and were instructed to classify the gender of each face. Participants were instructed to press their index finger to respond to female faces, and their middle finger to respond to male faces. Each participant was presented with a random order of male and female faces, with an equal proportion of male and female faces. The task was made up of twelve blocks, each consisting of a single emotion, with eight stimuli per block; each emotional condition block occurred four times in each run. Faces were displayed for 2 s, with each block lasting 16 s. Between each block, a central fixation cross was displayed for 16 s. We analysed the accuracy of gender classification (data not shown), and excluded one subject with performance worse than chance; the final analysis included 28 participants.

### Image acquisition and analysis

We acquired gradient-echo T2*-weighted images using a Siemens Avanto 1.5 T MRI scanner (32-channel head coil), with 36 slices per volume, and a slice gap of 1 mm (50% distance factor; 2 mm slices). We employed a highly optimized pulse sequence with a 32-channel head coil. The 32-channel coil improves SNR up to 3.5 times (Wiggins et al., 2006) compared to the standard 8 or 12 channel coil. In addition, susceptibility artefacts (i.e. signal dropout) in the regions of interest in our study are substantially increased at higher field strengths. Note, however, that the high spatial resolution provided by ultra-high field fMRI can mitigate the negative influences of physiological noise sources on ventral brain areas: previous work has reported a clear gain in percent signal change during facial emotion discrimination at 7 T (compared to 3 T) MRI (Sladky et al., 2013).

For each echo planar imaging (EPI) sequence, we used a 30° tilted sequence to minimize dropout in the ventral prefrontal cortex and amygdalae (O’Nions et al., 2011, Weiskopf et al., 2006). This acquisition protocol was developed by testing the optimal parameters to reduce susceptibility-induced BOLD sensitivity losses in the ventral prefrontal cortex (Weiskopf et al., 2006). Briefly, magnetic susceptibility varies greatly between tissue and air cavities and this variation can induce localised magnetic field gradients which interfere with the imaging field gradients causing distortion and loss of signal. The protocol optimized the phase encoded direction gradient polarity and slice orientation, such that signal losses due to induced field gradients within-slice, in the phase encoded direction, are small enough not to cause signal loss; and employed a "z-shimming" gradient prepulse to counteract signal loss from through-slice induced magnetic field gradients. However, this optimized sequence also gave us reduced coverage of the brain. For this reason, we include our second-level mask in the Supplemental materials (see Figure S1, A–C).

Echo time was 50 ms, repetition time per slice was 87 ms, slice thickness was 2 mm, and in-plane resolution was 2×2 mm. We acquired one fieldmap per subject per day with identical parameters to the EPI scans, and one five-minute magnetization-prepared rapid gradient-echo T1-weighted 1 mm isotropic anatomical scan for each subject.

EPI data were analysed using Statistical Parametric Mapping (SPM12; Wellcome Trust Centre for Neuroimaging, London, www.fil.ion.uck.ac.uk/spm) in Matlab R2015a. Due to the relatively long TR (3.132 s), we performed slice-time correction on all data to minimize differences over time in slice acquisition. After removing the first six volumes from each time series to allow for T1 equilibration, the remaining volumes were realigned to the seventh volume, coregistered to each subject's anatomical scan, normalized into standardized space (Montreal Neurological Institute template), and smoothed using an 8 mm full width at half maximum Gaussian kernel. Following the realignment stage, all image sequences were checked for movements greater than 1.5 mm or rotations greater than 1 degree in any direction – corrupted images were removed and replaced using interpolation. Following normalization, anatomical images were manually checked for artefacts.

Regressors of interest (see Table 1) were convolved with SPM's canonical synthetic hemodynamic response function time-locked to the onset of the corresponding event. We included six movement regressors of no interest in all subjects, and an error regressor of no interest only in subjects who made errors on the gender classification task. In the gender classification and emotion identification tasks, fixation periods constituted an implicit baseline. Using the general linear model, parameter estimate images were estimated for each regressor, and combined to create the primary contrast of interest for each task (for gender classification and emotion identification, faces vs fixation; for emotion matching, faces vs shapes).

Second-level analyses were conducted using the standard summary statistics approach to random effects analysis. We anticipated that we would identify amygdala activation (faces>fixation) and sgACC deactivation (fixation>faces) while viewing emotional faces. We identified the group-level peak voxel for each task's primary contrast, averaged across runs and days, in the left amygdala, right amygdala, and sgACC (see Table 2). We applied a cluster-forming threshold of *p*<0.001 (uncorrected) and report small volume corrected p-values for responses in the amygdalae and sgACC at the voxel- and cluster-levels in Table 2.

**Table 2:** fMRI results for activation to all faces in the relevant regions of interest.

**Region**	**Task**	**Coordinates (x,y,z)**	**Z-score**	**k (cluster size)**	***p*****value (cluster-level corrected)**	***p*****value (voxel-level corrected)**
sgACC	GC	6,35,5	5.52	152	<0.001	<0.001
	EI	6,38,2	4.84	80	<0.001	<0.001
	EM	−3,17,−7	5.12	43	0.001	0.001
Left amygdala	GC	−24 −7 −13	5.75	47	0.001	<0.001
	EI	−21,−4,−13	4.78	31	0.001	<0.001
	EM	−21,−7,−16	5.11	59	<0.001	<0.001
Right amygdala	GC	21,−4,−16	5.59	50	0.001	<0.001
	EI	21,−1,−13	4.58	28	0.001	<0.001
	EM	21,−4,−13	6.19	63	<0.001	<0.001
*Control region*						
Right FFA	GC	33, −37, −19	4.60	26	0.009	<0.001
	EI	39,−55,−25	5.96	53	0.001	<0.001
	EM	30,−46,−13	4.34	4	0.062	0.001

Cluster-forming threshold *p*=0.001 uncorrected, restricted to the relevant anatomical mask (sgACC, left amygdala; right amygdala; right fusiform gyrus); *p*-values are family-wise error small volume corrected (SVC). sgACC=subgenual anterior cingulate cortex. GC=gender classification; EI=emotion identification; EM=emotion matching. For the control region only, reliability analyses used the same coordinate (from a previous publication, (McKeeff and Tong, 2007)) for extracting activation across all tasks.

#### Functional ROI approach

There are two main approaches to calculating the within-subject test-retest reliability of fMRI: (1) extracting values from group-level peaks; (2) using *a priori* defined regions (Aron et al., 2006). We first employed approach 1: for the purpose of calculating reliability statistics, we created functional regions of interest (ROIs) separately for each task. We extracted parameter estimates for each subject from the left amygdala, right amygdala, and sgACC using a 4 mm sphere centered on each group-level peak voxel for the primary contrast from the relevant task. Anatomical masks from the PickAtlas toolbox were applied to confirm that the peak voxels fell within these structures; sgACC was defined as Brodmann Areas 24 and 25. Note that all of the contrasts used to identify activations (averaged across days and runs) are orthogonal to the analyses on which we make inference (effects of day and run; correlations between days and runs), thus avoiding non-independence error. We repeated this procedure for a second contrast, fearful>neutral faces, but failed to identify robust activation in these regions. We therefore do not report reliability statistics for this contrast.

We also calculated reliability statistics for a fourth region, the right fusiform face area (FFA), which served as a positive control. This allowed us to test the general reliability of our experimental design. To do this, we extracted parameter estimates for each subject from the right FFA using a 4 mm sphere centred on the right FFA coordinates reported in a previous publication (McKeeff and Tong, 2007) (MNI coordinates: 38, −43, −20). We repeated this procedure for the faces vs control contrast for each of the three tasks. For these analyses, we excluded subjects in whom coverage did not include the FFA (N=4); see Table 2 for group-level peak voxels within the fusiform gyrus, and Figure S1, D–F for the second-level masks.

#### Anatomical ROI approach

We also employed a second approach, using anatomically-defined ROIs to calculate reliability. For the amygdalae, we used masks from the PickAtlas toolbox. For the sgACC, we manually defined the volume on a representative participant's anatomical MRI using the borders and definition reported in previous probabilistic maps, which included several distinct cyto- and receptor-architectonic areas: 25, s24, s32, and the ventral portion of area 33 (Palomero-Gallagher et al., 2015).

### Reliability analysis

Subject-level parameter estimates (averaged across all voxels in ROIs) were extracted from SPM for each task, ROI and scanning session, and analysed with the Statistical Package for the Social Sciences 22 (http://www.ibm.com/analytics/us/en/technology/spss/). First, to test the effects of day and run on BOLD responses, we ran nine repeated measures analyses of variance (ANOVAs) on the extracted subject-level activations, for each task and ROI. Alpha=0.05 was set as the significance threshold.

To test our primary hypothesis, we calculated intra-class correlation coefficients (ICCs) for each ROI (left amygdala, right amygdala, sgACC, and our control region, right FFA) between the two scan days, using the average of the two runs on each day. We repeated this ICC analysis to test the reliability within each scan day (between the two runs). To test whether the first or second run of each day contributed particularly to reliability, we also calculated ICCs between the first and second runs separately between the two days (i.e., the reliability run 1 of day 1 and run 1 of day 2; and the reliability run 2 of day 1 and run 2 of day 2).

The ICC is a standard method to quantify the stability of measurements between test and retest sessions (Bennett and Miller, 2010). Most versions of the ICC are interpreted as a ratio of variances (Bartko, 1966), with ICCs approaching 1.0 indicating near-perfect agreement between the values of the test and retest measurements, and ICCs approaching 0 indicating little or no reliability. We assessed reliability using ICC(3,1), a 2-way mixed effects ICC, defined by Shrout and Fleiss (Shrout and Fleiss, 1979) as:

ICC(3,1)=BMS−EMS/BMS+(k−1)*EMS

Where BMS=between-subjects mean square; EMS=error mean square; k=number of repeated sessions (i.e., 2).

In our analyses, this form of the ICC indicates the correlation between BOLD response parameter estimates between sessions, and has been used in previous studies assessing the test-retest reliability of amygdala activation (Plichta et al., 2012, Johnstone et al., 2005, Sauder et al., 2013, Manuck et al., 2007, Lipp et al., 2014). Of note, in this ICC, the effect of measure (i.e., the scanner) is assumed to be fixed rather than random, while the effects of subjects are assumed to be random. We employ a “consistency” measure of ICC, rather than testing the absolute agreement between days or runs, due to the possibility that participants might habituate to the stimuli over time. Thus, a high ICC implies that the relative activations are consistent between days (i.e., the subjects with greater activation on day 1, relative to the rest of the group, are also those with greater activation on day 2). We report average measures ICC statistics as the calculation of parameter estimates in fMRI inherently involves averaging over many trials.

We adhere to a conventional interpretation of ICCs to quantify the degree of reliability: ICC<0.4 = poor reliability; 0.4–0.75=moderate to good reliability; >0.75=excellent reliability (Fleiss, 1986, Plichta et al., 2012). A negative ICC is usually interpreted as a reliability of zero (Bartko, 1976), since the theoretical limits of the ICC are 0–1 (although negative values can occur, when the within-groups variance exceeds the between group variance, this is outside the theoretical range (Lahey et al., 1983)). Statistical significance was of secondary concern in the reliability analysis, but we also report *p*-values for all reliable activations, and 95% confidence intervals for all ICCs, obtained from an *F*-test against the null hypothesis.

We calculated that 27 participants would be needed to achieve 80% power to detect an effect size (correlation) of *r*=0.5 (at alpha=0.05), with 0.5 chosen as it represents a potentially clinically meaningful degree of reliability.

## Results

### Average activation

**Fig. 1:**
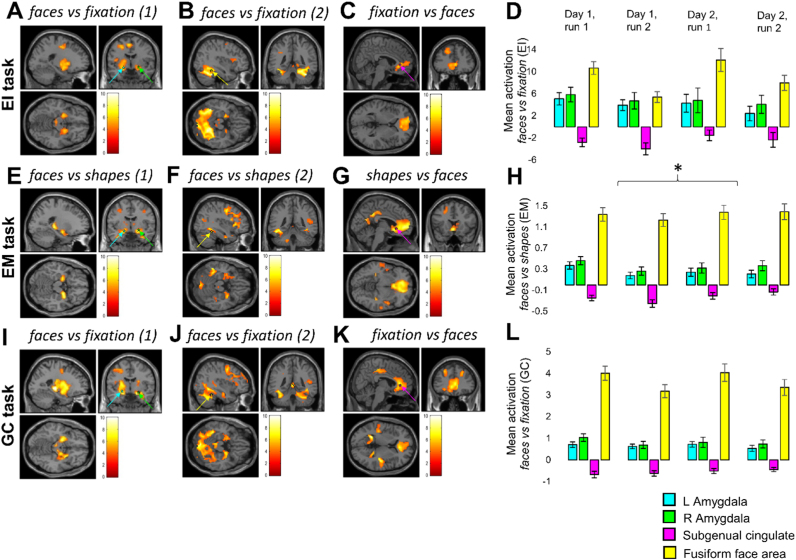
Whole-brain activation maps and parameter estimates for the three functionally-defined regions of interest (left and right amygdala, and subgenual anterior cingulate cortex, sgACC), and the comparison region, the right fusiform face area (FFA), for all runs (both days). Coloured arrows and stars indicate coordinates used in the analysis: cyan arrows correspond to peak activation in the left amygdala; green arrows to peak activation in the right; yellow arrows indicate the coordinate from a previous study (McKeeff and Tong, 2007) used for the FFA analysis; magenta arrows indicate peak activation in the sgACC. Images were thresholded at *p*<0.001 (uncorrected) and at the minimum cluster size surviving whole-brain cluster-level correction for each contrast; the heat bars indicate t-values. Please see Table 2 for statistics. A, E, I: *faces vs fixation (1)* and *faces vs shapes (1)* include all subjects; *faces vs fixation (2)* and *faces vs shapes (2)* (B, F, J) exclude the four subjects whose FFA was not included in the mask. Asterisk over the EM (*) bar chart depicts the only main or interaction effect of day or run: the effect of day on sgACC activation (*p*=0.045).

As expected, all tasks induced significant activation (surviving whole-brain correction for multiple comparisons) in the left and right amygdalae, and deactivation in the sgACC (Fig. 1 and Table 2). For each task, we conducted a two-way repeated measures ANOVA, for each functional ROI (left amygdala, right amygdala, and sgACC – average values across voxels in functional ROIs), examining the effects of day and run on activation. No main or interaction effects reached significance in the emotion identification task (Fig. 1D) or the gender classification task (Fig. 1L). In the emotion matching task (Fig. 1H), the sgACC deactivated more on the first day than on the second (*F*(1,28)=4.415, *p*=0.045), but there was no main effect of run or day-by-run interaction (both *p*>0.5), and no main effects or interactions in the other two ROIs reached significance. See Supplemental information for full statistics (Table S1), and Fig. 1 for whole-brain activation maps and parameter estimates for the three functional ROIs, with the control region, right FFA, presented for comparison purposes.

We repeated these analyses for the anatomically-defined ROIs. No main or interaction effects reached significance in the emotion identification task (Figure S2A; Table S1). There was a significant effect of day on sgACC response in the emotion matching task (*F*(1,28)=5.141, *p*=0.031) (Figure S2B), and gender classification task (*F*(1,26)=5.366, *p*=0.029) (Figure S2C): again, the sgACC deactivated more on the first day than on the second. There was also an interaction between day and run on sgACC response in the emotion matching task (*F*(1,28)=5.573, *p*=0.025): on the first day, the sgACC deactivated more on the second run; on the second day, it deactivated more on the first run (Figure S2B). No other main or interaction effects reached significance. See Supplemental information for full statistics (Table S1) and figures (Figure S2).

#### Post-hoc exploratory interactions with sex

We also examined whether participant sex interacted with measured activation in the amygdalae and sgACC in any of the three tasks. We ran these analyses for each task and region, first for the functionally-defined ROIs, examining the interaction between day and sex, between run and sex, and the three-way interaction between day, run, and sex (45 statistics in total: see Supplemental information Table S2). No regions or tasks showed any interaction with sex, with the exception of right amygdala activation in the emotion identification task, where there was a run-by-sex interaction (*F*(1,24)=5.401, *p*=0.029); however, most relevant for our analyses, there was no interaction with day, nor a three-way interaction between sex, run, and day.

We repeated this analysis for the anatomically-defined ROIs. In the emotion matching and gender classification tasks, no region showed any interaction with sex. In the emotion identification task, there was no interaction between sex and day, nor between sex, day, and run, but all three regions showed a run-by-sex interaction (left amygdala: *F*(1,25)=6.330, *p*=0.019, right amygdala: *F*(1,25)=7.921, *p*=0.009, sgACC: *F*(1,25)=4.796, *p*=0.038). For all three regions, this was driven by a reduction in activation (corresponding to an increase in deactivation in the sgACC) between run 1 and run 2 in male participants (on both days); female participants, by contrast, typically showed a small increase in activation (decrease in sgACC deactivation) between the two runs (data not shown).

### Within-subject reliability of activation (functional ROI analysis)

#### Between-day reliability

**Fig. 2:**
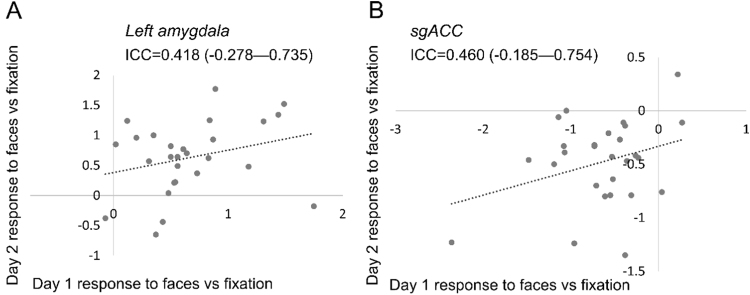
Distribution of parameter estimates for the left amygdala (A) and sgACC (B) in day 1 and day 2 for the gender classification task, for the main contrast (faces vs fixation). Note that for this task, parameter estimates are moderately correlated between scan days in both regions.

We examined the reliability of activation within each subject, across the two days. In most cases, between the first and second days (averaging across both runs on each day) the amplitude of BOLD responses showed poor reliability: the majority of ICCs we observed—seven out of nine reliability statistics— were well below 0.4 (see Table 3). In the gender classification task, both left amygdala (ICC=0.418, *p*=0.087, 95%CI=−0.278 to 0.735) and sgACC (0.460, *p*=0.061, 95%CI=−0.185 to 0.754) just exceeded the threshold for moderate reliability (0.4), see Fig. 2. We did not find any relationship between the stability of each participant's BOLD response between days (in any task or any region) and state or trait anxiety: see Supplemental analysis S1.

**Table 3:** Intra-subject reliability statistics for all tasks and contrasts (functional ROIs and control region).

**Task**	**Contrast**	**Reliability**	**LAmyg ICC (95% CI)**	**RAmyg ICC (95% CI)**	**sgACC ICC (95% CI)**	**FFA ICC (95% CI)**
**EI**	*Faces*>*fixation*	Between-day (both runs)	0.033 (−1.122 to 0.559)	−0.382 (−2.032 to 0.370)	−0.834 (−3.025 to 0.164)	0.547 (−0.470 to 0.804)*
		Between-day (run 1)	0.304 (−0.527 to 0.683)	0.211 (−0.731 to 0.641)	−0.753 (−2.847 to 0.201)	0.456 (−0.258 to 0.765)*
		Between-day (run 2)	0.365 (−0.393 to 0.711)	−0.124 (−1.466 to 0.488)	−0.793 (−2.935 to 0.183)	0.180 (−0.896 to 0.645)
		Within-day 1 (run 1/run 2)	−0.201 (−1.635 to 0.453)	−0.067 (−1.341 to 0.514)	0.025 (−1.248 to 0.533)	0.466 (−0.234 to 0.769)**
		Within-day 2 (run 1/run2)	0.172 (−0.818 to 0.622)	0.450 (−0.206 to 0.750)*	−0.193 (−1.618 to 0.456)	0.292 (−0.636 to 0.694)
**EM**	*Faces*>*fixation*	Between-day (both runs)	0.300 (−0.492 to 0.671)	−0.502 (−2.200 to 0.295)	−0.126 (−1.399 to 0.471)	0.830 (0.607 to 0.926)**
		Between-day (run 1)	0.121 (−0.872 to 0.587)	0.176 (−0.754 to 0.613)	0.124 (−1.394 to 0.472)	0.856 (0.666 to 0.938)**
		Between-day (run 2)	−0.478 (−2.149 to 0.306)	−0.283 (−1.733 to 0.397)	0.302 (−0.486 to 0.672)	0.765 (0.458–0.899)**
		Within-day 1 (run 1/run 2)	−0.240 (−1.641 to 0.418)	0.057 (−1.009 to 0.557)	0.346 (−0.393 to 0.693)	0.897 (0.763–0.956)**
		Within-day 2 (run 1/run2)	0.303 (−0.427 to 0.685)	0.321 (−0.446 to 0.681)	0.209 (−0.684 to 0.629)	0.903 (0.775–0.958)**
**GC**	*Faces*>*fixation*	Between-day (both runs)	0.418 (−0.278 to 0.735)*	0.231 (−0.687 to 0.650)	0.460 (−0.185 to 0.754)*	0.896 (0.759–0.955)**
		Between-day (run 1)	0.213 (−0.727 to 0.641)	−0.328 (−1.914 to 0.395)	0.473 (−0.156 to 0.760)*	0.854 (0.662–0.937)**
		Between-day (run 2)	0.574 (0.066 to −0.806)*	0.552 (0.018–0.796)*	0.041 (−1.104 to 0.563)	0.902 (0.774–0.958)**
		Within-day 1 (run 1/run 2)	0.234 (−0.681 to 0.651)	−1.190 (−3.806 to 0.002)	0.213 (−0.727 to 0.641)	0.855 (0.664–0.937)**
		Within-day 2 (run 1/run2)	0.671 (0.278 to 0.850)*	0.770 (0.495–0.895)*	−0.013 (−1.222 to 0.538)	0.948 (0.880–0.977)**

For each functionally-defined region of interest as well as our control region (the right fusiform face area, FFA, defined using a 4 mm sphere around coordinates from a previous publication (McKeeff and Tong, 2007)), the within-subject reliability of between-day and within-day activation of each task. Between-day reliability is reported averaging across both runs, and for each run separately. Within-day reliability is reported for day 1 and day 2 separately. A negative ICC is interpreted as indicating a reliability of zero (Bartko, 1976). GC=gender classification; EI=emotion identification; EM=emotion matching; LAmyg=left amygdala; RAmyg=right amygdala; sgACC=subgenual anterior cingulate cortex; ICC=inter-class correlation coefficient; CI=confidence interval.

* ICC>0.4.

** ICC>0.75.

Separately examining the reliability of responses during the first and second runs of each task between days again revealed no instances of reliability in the emotion identification task, or in the emotion matching task, but three (out of 18) instances of moderate-to-good reliability in the gender classification task: reliability exceeded 0.4 in the sgACC between the first runs of each day (ICC=0.473, *p*=0.054, 95%CI=−0.156 to 0.760), and in the left (ICC=0.575, *p*=0.017, 95%CI=0.066–0.806) and right amygdala (ICC=0.552, *p*=0.023, 95%CI=0.018–0.796) in the second runs of each day.

There were no other reliable activations on any of the three tasks, either averaging across runs or examining them separately.

#### Within-day reliability

Most tasks also showed poor within-day reliability (i.e., the reliability of activation in run 1 versus run 2 on the same day). There were three exceptions (of 18 tests), all on the second day: in the emotion identification task, the right amygdala showed moderate reliability (ICC=0.450, *p*=0.067, 95%CI=−0.206 to 0.750), and in the gender classification task, both left (ICC=0.671, *p*=0.003, 95%CI=0.278–0.850) and right (ICC=0.770, *p*<0.001, 95%CI=0.495–0.895) amygdalae showed very good reliability. No other task evoked reliable within-day responses in any of the three ROIs.

#### Reliability of control region

We repeated these analyses for our control region, the right FFA (see Table 3). In almost all cases (13/15 tests), the right FFA showed at least moderately reliable activations, both between and within-day. In the two block design tasks, emotion matching and gender classification, the right FFA showed excellent reliability in every instance (all ICC>0.75; all *p*<0.001), in both within-day and between-day analyses. In the event-related emotion identification task, the right FFA showed moderate-to-good reliability (ICC>0.4) between days overall, as well as between days in the first runs, and across runs on the first day. However, in this task we found poor reliability between the second runs of each day (ICC=0.180, *p*=0.319, 95%CI=−0.896 to 0.645), and within the second day, across runs (ICC=0.292, *p*=0.207, 95%CI=−0.636 to 0.694).

### Within-subject reliability of activation (anatomical ROI analysis)

#### Between-day reliability

For our secondary reliability analysis, we used the values extracted from the anatomical ROIs to examine the reliability of activation within each subject, across the two days. See Table 4 for full statistics. In this case, between the first and second days (averaging across both runs on each day) the amplitude of BOLD responses showed low reliability in six out of nine analyses. Two exceptions were in the sgACC: for the emotion identification task (ICC=0.564, *p*=0.019, 95%CI=0.043–0.801) and for the gender classification task (ICC=0.577, *p*=0.016, 95%CI=0.073–0.807); one was in the left amygdala, for the emotion matching task: (ICC=0.432, *p*=0.07, 95%CI=−0.210 to 0.733).

**Table 4:** Intra-subject reliability statistics for all tasks and contrasts (anatomical ROI).

**Task**	**Contrast**	**Reliability**	**LAmyg ICC (95% CI)**	**RAmyg ICC (95% CI)**	**sgACC ICC (95% CI)**
**EI**	*Faces*>*fixation*	Between-day (both runs)	0.190 (−0.777 to 0.631)	−0.127 (−1.473 to 0.486)	0.564 (0.043–0.801)*
		Between-day (run 1)	0.227 (−0.696 to 0.648)	0.093 (−0.991 to 0.586)	0.297 (−0.543 to 0.680)
		Between-day (run 2)	0.175 (−0.811 to 0.624)	−0.152 (−0.862 to 0.613)	0.524 (−0.045 to 0.783)*
		Within-day 1 (run 1/run 2)	0.235 (−0.678 to 0.651)	0.002 (−1.189 to 0.545)	0.445 (−0.217 to 0.747)*
		Within-day 2 (run 1/run2)	0.214 (−0.724 to 0.642)	0.523 (−0.047 to 0.783)*	−0.124 (−1.466 to 0.488)
**EM**	*Faces*>*fixation*	Between-day (both runs)	0.432 (−0.210 to 0.733)*	−0.141 (−1.141 to 0.464)	0.326 (−0.435 to 0.684)
		Between-day (run 1)	0.090 (0.939 to 0.573)	−0.027 (−1.187 to 0.518)	0.445 (−0.197 to 0.744)*
		Between-day (run 2)	0.095 (−0.995 to 0.581)	0.013 (−1.102 to 0.537)	0.139 (−0.834 to 0.596)
		Within-day 1 (run 1/run 2)	0.053 (−1.018 to 0.555)	−0.323 (−1.818 to 0.379)	0.659 (0.274 to 0.840)*
		Within-day 2 (run 1/run2)	0.148 (−0.815 to 0.600)	0.137 (−0.837 to 0.595)	0.353 (−0.378 to 0.696)
**GC**	*Faces*>*fixation*	Between-day (both runs)	0.069 (−1.043 to 0.576)	0.226 (−0.699 to 0.647)	0.577 (0.73–0.807)*
		Between-day (run 1)	0.079 (−0.121 to 0.580)	0.112 (−1.440 to 0.493)	0.317 (−0.498 to 0.689)
		Between-day (run 2)	0.289 (−0.559 to 0.676)	0.205 (−0.743 to 0.638)	0.205 (−0.743 to 0.638)
		Within-day 1 (run 1/run 2)	0.328 (−0.474 to 0.694)	0.256 (−0.632 to 0.661)	0.519 (−0.055 to 0.781)*
		Within-day 2 (run 1/run2)	0.811 (0.585 to 0.914)**	0.344 (−0.440 to 0.701)	−1.007 (−3.405 to 0.085)

For each anatomically-defined region of interest, the reliability of between-day and within-day activation of each task. Between-day reliability is reported averaging across both runs, and for each run separately. Within-day reliability is reported for day 1 and day 2 separately. A negative ICC is interpreted as indicating a reliability of zero (Bartko, 1976). GC=gender classification; EI=emotion identification; EM=emotion matching; LAmyg=left amygdala; RAmyg=right amygdala; sgACC=subgenual anterior cingulate cortex; ICC=inter-class correlation coefficient; CI=confidence interval.

* ICC>0.4.

** ICC>0.75.

Separately examining the reliability of responses during the first and second runs of each task between days also revealed low reliability in 16 out of 18 analyses, with two exceptions, both in the sgACC: the reliability of the second runs of the emotion identification task (ICC=0.524, *p=*0.032, 95%CI=−0.045 to 0.783) and the first runs of the emotion matching task (ICC=0.445, *p*=0.066, 95%CI=−0.197 to 0.744).

#### Within-day reliability

The majority of comparisons showed poor within-day reliability (i.e., the reliability of activation in run 1 versus run 2 on the same day). There were five exceptions (out of 18 analyses): the sgACC on the first day in all three tasks: the emotion identification task (ICC=0.445, *p*=0.07, 95%CI=−0.217 to 0.747), the emotion matching task (ICC=0.659, *p*=0.003, 95%CI=0.274—0.840), and the gender classification task (ICC=0.519, *p*=0.034, 95%CI=−0.055 to 0.781), the left amygdala on the second-day in the gender classification task (ICC=0.811, *p*<0.001, 95%CI=0.585–0.914), and the right amygdala on the second day in the emotion identification task (ICC=0.523, *p*=0.032, 95%CI=−0.047 to 0.783).

## Discussion

We observed surprisingly low within-subject reliability of three putative fMRI biomarkers, the amygdalae and sgACC response to emotional faces. We identified just one instance of moderate between-day reliability (averaged across both runs) that was consistent between functional and anatomical ROI approaches – in the sgACC on the gender classification task. However, the FFA showed good-to-excellent between-day reliability in every task. All but one of 18 analyses also showed reliable within-day activity in the FFA. By contrast, only three (using the functional approach) and five (using the anatomical approach) showed at least moderate within-day reliability in the amygdalae and sgACC, with most found on the second day.

There were few substantive differences between the two ROI approaches: the anatomical ROI approach yielded ten instances of at least moderate reliability (out of 45 analyses) and the functional ROI approach yielded eight (see Table 3, Table 4). In three cases, the same analyses were reliable using both approaches: the overall between-day reliability of the sgACC and the within-day reliability (on day 2) of the left amygdala during the second run (both in the gender classification task); and the within-day reliability (on day 2) of the right amygdala (in the emotion identification task). The remaining analyses differed between the two approaches. The most notable difference between the approaches was that using the functional ROI approach, almost all instances of reliability occurred in the gender classification task; the anatomical ROI approach produced reliability scores that were more evenly distributed across the tasks. The anatomical approach also showed some regional specificity: seven out of ten instances of reliability were found in the sgACC. Nonetheless, overall both approaches revealed generally low reliability.

These findings have practical implications for studies hoping to use BOLD responses in these regions as treatment biomarkers in psychiatry. Our results suggest that many of the task-evoked responses assumed to be stable within individuals may not in fact be viable fMRI biomarkers, at least not using these three common paradigms. They also suggest potentially important task and regional differences between functional and anatomical ROI approaches.

Several studies have reported low reliability of amygdala BOLD responses between two scanning days (Johnstone et al., 2005, Sauder et al., 2013, Lipp et al., 2014), though it is unexpected that even within the same scanning session, amygdala response was not particularly reliable. We are also (to our knowledge) the first to specifically test the reliability of BOLD responses in the sgACC, a region frequently identified in treatment biomarker studies in depression (Keedwell et al., 2009, Keedwell et al., 2010, Chen et al., 2007, Davidson et al., 2003). We suggest that the sgACC and left amygdala response to emotional faces may be reliable across days under certain conditions, which differ depending on the task and ROI approach used. However, even in these circumstances, we found low-to-moderate reliability, just exceeding our ICC threshold of 0.4, but not ever meeting the threshold for excellent reliability (ICC>0.75). Certainly in contrast to the BOLD response of the FFA, which was ‘excellent’ (>0.75) in 10/15 analyses, the between-day reliability of the amygdalae and sgACC seems markedly weaker.

It bears mentioning that our anatomical and functional approaches produced, in most cases, different instances of reliability, with the anatomical approach yielding slightly higher ICCs overall. Nevertheless, in our results, even the anatomical technique did not yield high reliability in all, or even most, instances. Overall, our findings do not provide strong grounds for optimism for the use of amygdala or sgACC responses to emotional faces as treatment biomarkers (at least on their own: the potential for more complex fMRI biomarkers, for example using pattern classification techniques, remains (Fu et al., 2008a)). Our findings have significant practical implications for the design and analysis of studies employing putative fMRI biomarkers: researchers are encouraged to consider region, task, and ROI selection approaches to develop a useful fMRI biomarker.

### Limitations

We selected *a priori* regions to test that have been strongly implicated in neurobiological studies of depression and its treatment; these have often been proposed as potential treatment biomarkers. It is quite possible that other regions—for example, the dorsolateral prefrontal cortex, also implicated in depression (Koenigs and Grafman, 2009)—might show more consistent within-subject reliability than the amygdalae or sgACC. Indeed, our control analysis of the right FFA indicates that this region is extremely reliable.

Our study only tested the reliability of these three regions in a non-clinical sample of healthy, young individuals. Future studies could expand our finding to relevant clinical samples, directly testing the reliability of putative treatment biomarkers in a population with neuropsychiatric symptoms. This strategy may yield greater reliability, as observed, for example, in patients with multiple sclerosis relative to healthy controls (Bosnell et al., 2008) (note the reverse has also been shown: regions where healthy controls, but not patients with schizophrenia, show reliable activity (Manoach et al., 2001)). This could be particularly important with respect to selection of useful fMRI contrasts: in our study, as in some previous experiments (Deeley et al., 2006, Guyer et al., 2008), we failed to find amygdala activation in the fearful-neutral faces contrast, possibly as a result of habituation to the emotional face stimuli. However, it has also been suggested that individual differences in anxiety could account for the degree of amygdala activation to fearful faces (Calder et al., 2011). Replicating our study in a clinical population would better clarify which contrasts are most reliable in neuropsychiatric populations, and better guide future development of fMRI biomarkers. Furthermore, subclinical differences in anxiety and mood state between the scan dates could account for unreliable neural responses to the emotional stimuli. Future research should examine the effect of short term (state) and longer-term (trait) measures of mood and anxiety on the reliability of BOLD responses in the amygdalae and sgACC.

### Future directions

Our results describe an important issue to consider for fMRI treatment biomarkers, but whether (task-related) fMRI is the optimal choice for neuroimaging biomarkers still remains to be determined. Other putative neuroimaging biomarkers have been suggested: resting state connectivity has been shown to be highly reliable, and is associated with response to transcranial magnetic stimulation (Salomons et al., 2014); structural MRI, also highly reliable, when analysed using machine learning techniques, has successfully predicted individual response to electroconvulsive therapy (Redlich et al., 2016). Normalization of sgACC metabolism by antidepressant medication, measured with positron emission tomography (PET), has also been proposed as a prerequisite for symptom remission (Mayberg, 2003). Clinical response to fluoxetine at six weeks was associated with metabolic decreases in regions including the sgACC, detected after only one week of treatment; sgACC changes were absent in fluoxetine non-responders (Mayberg et al., 2000). In a separate study, changes in sgACC metabolism differentiated patients who responded to CBT from those who responded to venlafaxine (Kennedy et al., 2007).

Although structural MRI and PET are not dependent on the variability of an individual's BOLD response, making these putative biomarkers potentially more reliable, fMRI is less expensive and far more commonly-used than PET. Thus, the potential use of fMRI as a treatment biomarker in depression is more practical than for PET, providing the incentive for our investigation. As with any methodology, development of a useable biomarker must ensure test-retest reliability at the level of the individual: thus, our study might guide future work towards establishing tasks that evoke more reliable amygdala or sgACC activation. However, it might also encourage psychiatry researchers to look outside the most frequently-proposed regions for treatment biomarkers to those with more reliable BOLD responses.

A final suggestion for future research lies in the investigation of variability itself as a useful marker of susceptibility to emotional disorders. Habituation of the amygdala to emotional cues has previously been shown to be a marker of vulnerability to depression (Siegle et al., 2007, Siegle et al., 2002); low and high anxious subjects also show differences in amygdala habituation (Sladky et al., 2012). Measures of stability could therefore contain key information about individual differences, which could be employed in clinical studies. This possibility is strengthened by results from a similar test-retest experiment that found improved amygdala reliability (particularly in the right amygdala) when analysing the habituation, rather than amplitude, of the BOLD response (Plichta et al., 2012). Future clinical studies (as well as test-retest studies) would benefit from analysing regional habituation as well as amplitude in the search of robust, reliable fMRI biomarkers. This approach might yield more consistent reliability than reported in our study (Phillips et al., 2001). The search for fMRI biomarkers, then, requires careful scrutiny of region, task, measure, and analysis, all of which influence how stable the BOLD response appears.

## Funding

This work was supported by a Wellcome Trust Senior Investigator Award (to JPR: 101798/Z/13/Z). OJR is funded by a Medical Research Council Career Development Fellowship (MR/K024280/1). CJC was funded by a UCL Grand Challenges PhD Studentship. CLN is funded by a Brain Research Trust PhD Studentship.
