# Supplementary material for: Unreliability of putative fMRI biomarkers during emotional face processing
Source: Neuroimage. 2017 Aug 1;156:119–27. doi: 10.1016/j.neuroimage.2017.05.024 (PMC5553850; doi:10.1016/j.neuroimage.2017.05.024)
Supplement: Supplementary file 1 — Supplementary material [file mmc1.docx]

**Supplemental Information**

**
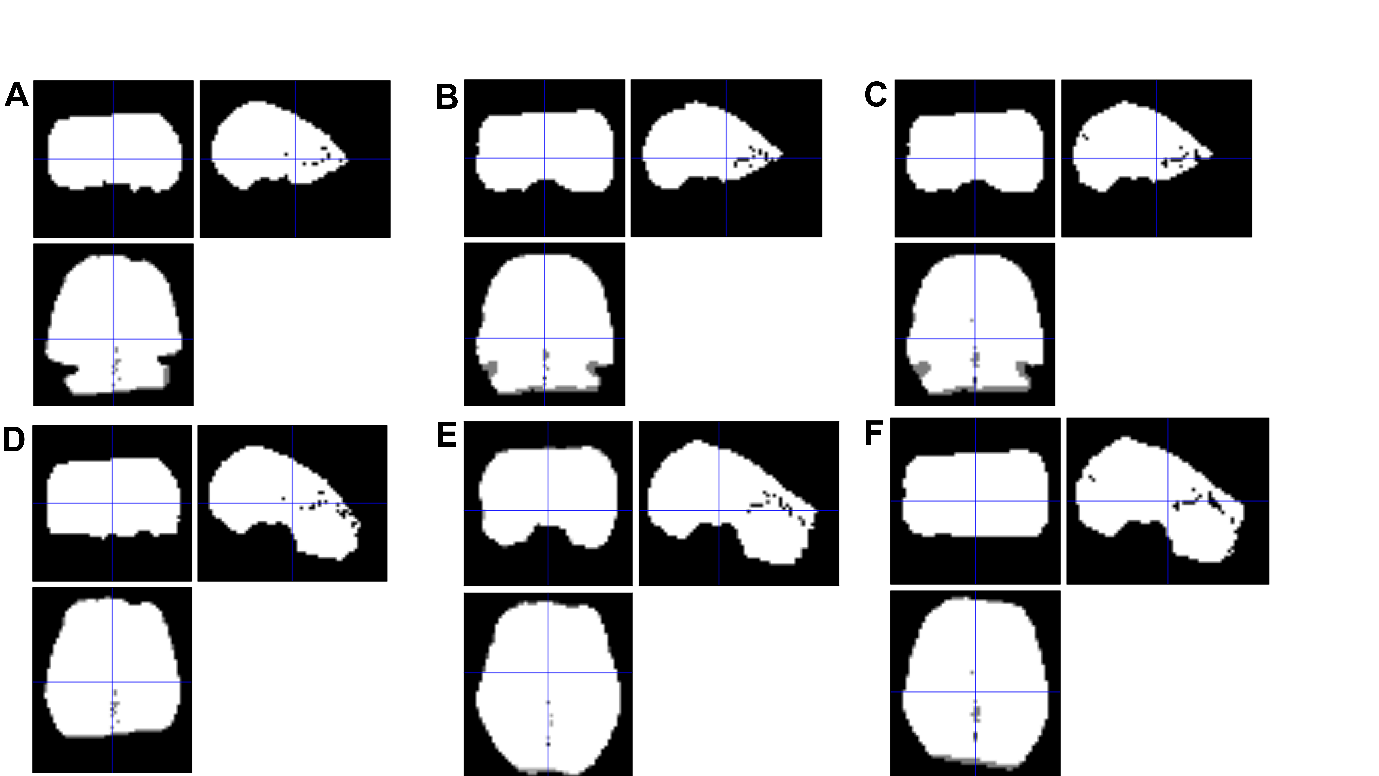
**

**Figure S1.** Second-level mask for each task for the primary analyses (A-C) and the control analyses of FFA (D-F). For the reliability analysis of the amygdalae and subgenual anterior cingulate cortex, note the reduced area due to incomplete coverage for the emotion identification (A), emotion matching (B), and gender classification (C) tasks. However, all three regions of interest (right amygdala, left amygdala, and subgenual anterior cingulate cortex) were covered in all subjects. For our control analyses only, we excluded four subjects whose fusiform face area (FFA) was not included in their masks. Note the inclusion of the right FFA, our control region, in the masks for the emotion identification (D), emotion matching (E), and gender classification (F) tasks.

**Table S1.** *Results of repeated measures ANOVAs examining effects of day and run on amygdala and sgACC activation*

| **Task** | **Anatomical region** | **Main effect: day**  *F* statistic | *p*-value | **Main effect: run**  *F* statistic | *p*-value | **Interaction effect: day*run**  *F* statistic | *p*-value |
| --- | --- | --- | --- | --- | --- | --- | --- |
| EI | *Left amygdala* | 0.801 | 0.379 | 1.021 | 0.321 | 0.132 | 0.719 |
| (1) | *Right amygdala* | 0.181 | 0.674 | 0.267 | 0.610 | 0.026 | 0.874 |
|  | *sgACC* | 1.560 | 0.223 | 1.117 | 0.300 | 0.020 | 0.889 |
| EM | *Left amygdala* | 0.569 | 0.457 | 3.291 | 0.058 | 1.012 | 0.323 |
| (1) | *Right amygdala* | 0.035 | 0.853 | 0.725 | 0.402 | 2.881 | 0.101 |
|  | *sgACC* | 4.415 | 0.045* | 0.268 | 0.609 | 0.179 | 0.676 |
| GC | *Left amygdala* | 0.082 | 0.777 | 1.385 | 0.250 | 0.390 | 0.538 |
| (1) | *Right amygdala* | 0.186 | 0.670 | 1.537 | 0.226 | 0.634 | 0.433 |
|  | *sgACC* | 2.432 | 0.131 | 0.245 | 0.625 | 0.003 | 0.956 |
| EI | *Left amygdala* | 0.365 | 0.551 | 0.784 | 0.384 | 0.006 | 0.939 |
| (2) | *Right amygdala* | 0.063 | 0.804 | 0.468 | 0.500 | 0.697 | 0.412 |
|  | *sgACC* | 0.208 | 0.652 | 0.220 | 0.643 | 1.554 | 0.224 |
| EM | *Left amygdala* | 0.019 | 0.891 | 0.674 | 0.419 | 1.682 | 0.205 |
| (2) | *Right amygdala* | 0.014 | 0.905 | 0.015 | 0.905 | 4.049 | 0.054 |
|  | *sgACC* | 5.141 | 0.031* | 0.491 | 0.489 | 5.573 | 0.025* |
| GC | *Left amygdala* | 0.657 | 0.425 | 2.774 | 0.108 | 0.034 | 0.855 |
| (2) | *Right amygdala* | 0.037 | 0.849 | 2.076 | 0.162 | 0.036 | 0.851 |
|  | *sgACC* | 5.366 | 0.029* | 1.246 | 0.274 | 0.018 | 0.895 |

(1) Extracted from functional ROIs (2) extracted from anatomical ROIs. sgACC = subgenual anterior cingulate cortex; GC = gender classification task; EI = emotion identification task; EM = emotion matching task. * *p*<0.05. Degrees of freedom: EI (1,26); EM (1,28); GC (1,26).

**
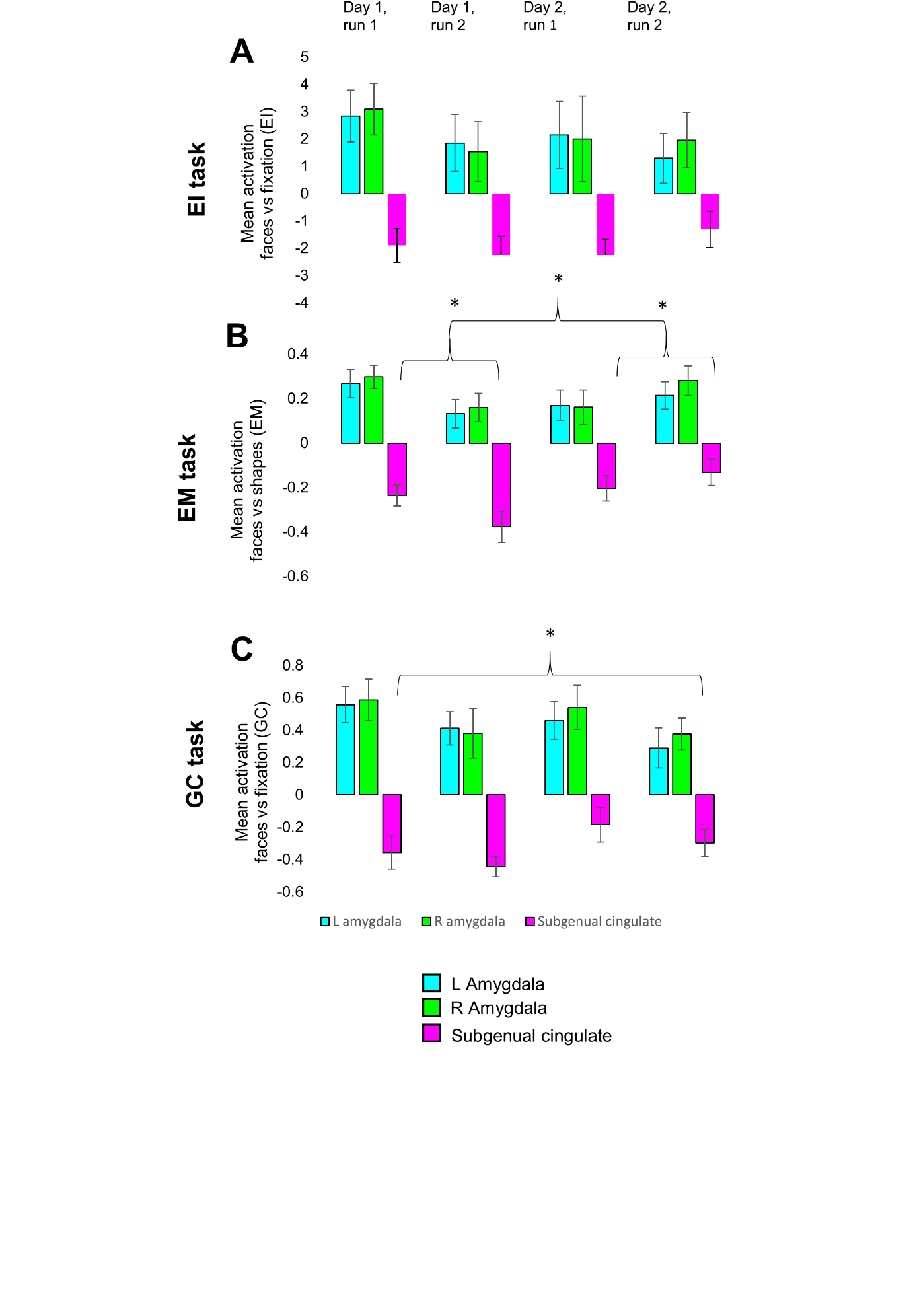
**

**Figure S2.** Parameter estimates for the primary contrast in anatomically-defined regions of interest. Parameter estimates for amygdalae and sgACC in the emotion identification (EI) task (A); parameter estimates for amygdalae and sgACC in the emotion matching task (B); and parameter estimates for amygdalae and sgACC in the gender classification task (C). Error bars indicate the standard error of the mean. Asterisks (*) depict the significant effect of day in the GC task, and the significant effect of day, and significant interaction between day and run in the EM task.

**Table S2.** *Results of repeated measures ANOVAs examining the interaction between sex and the effects of day and run on amygdala and sgACC activation*

| **Task** | **Anatomical region** | **Interaction effect: day*sex**  *F* statistic | *p*-value | **Interaction effect: run*sex**  *F* statistic | *p*-value | **Interaction effect: day*run*sex**  *F* statistic | *p*-value |
| --- | --- | --- | --- | --- | --- | --- | --- |
| EI | *Left amygdala* | 0.084 | 0.771 | 3.162 | 0.088 | 0.650 | 0.428 |
| (1) | *Right amygdala* | 1.669 | 0.208 | 5.401 | 0.029 * | 4.138 | 0.053 |
|  | *sgACC* | 0.476 | 0.496 | 2.389 | 0.135 | 0.550 | 0.465 |
| EM | *Left amygdala* | 0.012 | 0.914 | 0.889 | 0.354 | 0.094 | 0.762 |
| (1) | *Right amygdala* | 0.111 | 0.741 | 0.003 | 0.955 | 0.285 | 0.598 |
|  | *sgACC* | 0.773 | 0.387 | 0.027 | 0.870 | 0.795 | 0.381 |
| GC | *Left amygdala* | 0.308 | 0.584 | 0.018 | 0.894 | 0.169 | 0.685 |
| (1) | *Right amygdala* | 0.116 | 0.737 | 0.103 | 0.751 | 4.185 | 0.051 |
|  | *sgACC* | 0.729 | 0.402 | 0.524 | 0.476 | 0.321 | 0.576 |
| EI | *Left amygdala* | 0.830 | 0.371 | 6.330 | 0.019* | 0.229 | 0.636 |
| (2) | *Right amygdala* | 1.185 | 0.287 | 7.921 | 0.009* | 1.304 | 0.264 |
|  | *sgACC* | 3.222 | 0.085 | 4.796 | 0.038* | 0.167 | 0.686 |
| EM | *Left amygdala* | 0.086 | 0.772 | 1.168 | 0.289 | 0.565 | 0.459 |
| (2) | *Right amygdala* | 0.285 | 0.598 | 0.942 | 0.340 | 1.051 | 0.314 |
|  | *sgACC* | 1.224 | 0.278 | 0.046 | 0.832 | 3.950 | 0.057 |
| GC | *Left amygdala* | 0.215 | 0.647 | <0.001 | 0.995 | 2.597 | 0.120 |
| (2) | *Right amygdala* | 1.967 | 0.173 | 0.256 | 0.617 | 3.309 | 0.081 |
|  | *sgACC* | 2.554 | 0.123 | 0.797 | 0.380 | 0.415 | 0.525 |

(1) Extracted from functional ROIs (2) extracted from anatomical ROIs. sgACC = subgenual anterior cingulate cortex; GC = gender classification task; EI = emotion identification task; EM = emotion matching task. * *p*<0.05. Degrees of freedom: EI (1,26); EM (1,28); GC (1,26).

**Analysis S1**

Our healthy control sample presented with low anxiety levels, as measured by the State-Trait Anxiety Inventory (STAI); with a mean of 29.7 (SD=6.4) for state anxiety and 33.9 (6.8) for trait anxiety. We ran supplemental Pearson correlation analyses to examine the effects of state and trait anxiety level on the change in activation between days (Day 2 – Day 1) in each of the tasks, for each region (18 tests in total). This difference did not correlate with either state or trait anxiety in any region for any of the tasks (all *p*>0.1).
